# Changes of the prescription of hormone therapy in menopausal women: An observational study in Taiwan

**DOI:** 10.1186/1471-2458-7-56

**Published:** 2007-04-17

**Authors:** Weng-Foung Huang, Yi-Wen Tsai, Fei-Yuan Hsiao, Wen-Chun Liu

**Affiliations:** 1 Institutes of Health and Welfare Policy, National Yang-Ming University, Taipei, Taiwan; 2 Division of Health Policy Research, National Health Research Institutes, Miaoli, Taiwan; 3 Division of Health and Welfare Policy Management, Institutes of Public Health, National Yang-Ming University, Taipei, Taiwan; 4 Taiwan Joint Commission on Hospital Accreditation, Taipei, Taiwan

## Abstract

**Background:**

To evaluate the impact of the 2002 Women's Health Initiative (WHI) study results on the prescription of menopausal hormone therapy (MHT) to treat menopause-related symptoms in Taiwan.

**Methods:**

This retrospective study participant data collected from women interviewed in 2001 Taiwan's National Health Interview Survey (NHIS) and the National Health Insurance (NHI) outpatient claims for women being treated for menopause-related symptoms. We compared prescriptions made for MHI to women seeking outpatient treatment for menopause-related symptoms before and after the publication of the 2002 WHI to study its effect of prescription behavior in Taiwan. There was one dichotomous outcome variable, which was whether MHT was prescribed or not in an outpatient visit to treat menopause-related symptoms.

**Results:**

Our study included 504 women 45 years old or above whose outpatient visits for menopause-related symptoms were covered by National Health Insurance in 2002. In total, these 504 women made 2549 outpatient visits to be treated for these symptoms. The proportion of outpatient visits in which MHT was prescribed dropped from 83.0% (n = 1,155) before WHI to 73.0% (n = 844) after WHI. We found a decrease in likelihood that women would be prescribed MHT for menopause-related symptoms after the release of the WHI report (OR = 0.36, 95%CI = 0.25 to 0.52, p < 0.05). Gynecologists and obstetricians are more likely to prescribe MHT than physicians with other medical specialties (5.34; 95%CI = 3.45 to 8.26, p < 0.05). Women with college level educations or higher became less likely to be prescribed MHT (Model 2; OR 0.30; 95% CI 0.11–0.83), and academic medical centers became less likely to prescribe MHT than other medical care institutions (Model 3; OR 0.15; 95% CI 0.34–0.63).

**Conclusion:**

The WHI report caused a substantial decline in the use of MHT to treat menopause-related symptoms in Taiwan. It was found to exert most of its influence in patients with higher educations, physicians with specialties other than gynecologists and obstetricians, and academic medical centers.

## Background

Menopausal hormone therapy (MHT), estrogen with or without progestin, has been commonly used to treat symptoms of menopause and prevent chronic conditions such as cardiovascular disease and osteoporosis since the late 1960s [1]. Evidence on the potential risks and benefits of combined estrogen/progestin has slowly accumulated and has suggested that the combination of the two acts differently than estrogen alone [2-4]. Evidence from secondary prevention trials and observational studies has also shown that using combined estrogen/progestin therapy comes with an increased risk of coronary heart disease [5,6].

In 2002, the Women's Health Initiative (WHI) report [7] demonstrated that the risks outweighed the benefits for women undergoing continuous estrogen and progestin treatment [5]. The WHI report findings prompted the U.S. Food and Drug Administration to require new warning labels be placed on all estrogen products [8], and prompted the U.S. Preventive Services Task Force to recommend that estrogen and progestin not be used routinely in the prevention of chronic post-menopausal conditions [9]. Although WHI results may not apply to other types of estrogen and progesterone therapies, different dosages, and various methods of administration, the results were, nevertheless, significant enough to lead to a substantial decline in the use of MHT by many postmenopausal women [10-17].

Consequently, the WHI report affected drug approval policy. The FDA (U.S.) now only approves estrogen for the relief of menopause symptoms alone regardless of formulation, dosage, or route of administration. Furthermore, it has been recommended that only women at a significant risk of osteoporosis consider hormone therapy, at recommended dosage decreased from 0.625 mg conjugated estrogens to 0.3 mg [36]. Because the impact of the WHI report may demonstrate that what some studies have claimed- the dissemination and availability of health care information influence consumer perception and demand for medical care [10,18-20], we believe that a closer examination of the effect of WHI report on the use of MHT treatment might lead to a better understanding of the role of "information" in the demand and supply of medical services.

Certain consumer characteristics including age, educational level, working status, and marital status, influence whether or not a woman seeks and accepts MHT [1,15,21-24]. Also, possession of information or knowledge, largely obtained through medical and social contacts, has a great influence on the acceptance of MHT [[15,23,25], and [26]]. Furthermore, the opinion of a woman's health care provider has also been found to strongly influence her decision to undergo MHT [27].

Which medical interventions health care providers decide to use depend on their access to technical information, recommended procedures and regimen, and reported "uncertainties" about the effectiveness of the intervention [28]. Incomplete information, as being one of the main reasons, is often cited that physicians vary in how they treat a particular medical condition and in their patterns of practice [29]. Preference of medical intervention can also depend on medical specialty of the physician. Women treated by gynecologists have been reported to be 2.6 times more likely to receive MHT than women being treated by family physicians [30], which is understandable in light of the finding that gynecologists have a more favorable attitude toward MHT as a preventive measure than other physicians [31-33].

Forty to 60-year-old women in Taipei, Taiwan increased their use of MHT from 8.8% to 19% between 1991 and 1997 [34]. Most of these women were encouraged to seek this therapy by their social contacts, medical professionals and the media. The government even had a television advertisement promoting MHT in 1999. However, four months after the release of WHI report, the government formed an ad hoc committee that later announced new guidelines for the use of MHT, though the television promotion of MHT was withdrawn much earlier. [35].

With regard to WHI report's impact over physician and patients, it is not clear whether it affected their willingness to prescribe or use MHT in Taiwan. Using the results of a national health interview survey administered between August 2001 and January 2002 and linking them with the National Health Insurance (NHI) outpatient claims for women with menopausal conditions, we examined the changes of prescription of MHT to treat menopausal outpatients in Taiwan before and after the publication of WHI report as well as to what extent certain patient or health care provider characteristics influenced the decision to use MHT.

## Methods

### Data source

Data was drawn from two sources: the 2001 National Health Interview Survey (NHIS) and the NHI outpatient records of interviewees who consented to the linking of their personal information from the two sources. The data from the 2001 NHIS was collected between August 2001 and January 2002. The survey, using a stratified multistage sampling design with the probability of selection proportional to population size, included a representative sample of Taiwan's population of 23 million people and an over sampling of residents in the mountain regions and Taiwan's various offshore islands. The primary sample units, segmented based on their level urbanization levels defined by their socioeconomic and demographic characteristics, were 368 townships throughout Taiwan and outlying islands, 12 districts in the Taipei Municipality, and 11 districts in the Kaohsiung Municipality. All family members of each sampled household were included in our sample. NHI data were linked with NHIS data by personal identification number only if the respondents in the survey signed the informed consent.

The survey included 23,473 individuals (5798 households), representing Taiwan's general population. From this pool, we selected 3439 women 45 years old or above. After excluding 564 women who did not consent to the linkage of their survey data with their NHI data (21 with missing data), we were left with 2875 participants. Those who refused to participate were more likely to be older (aged 65 years or above, p < 0.0005), lack formal education (p < 0.0005), be unmarried (p < 0.05), be unemployed (p < 0.0005), or lack personal income (p < 0.0005) (Table 1).

**Table 1:** Characteristics of females aged 45 years or above, with and without informed consent.

	**Total^a^ (n = 3439)**		**No consent (n = 543)**		**Consent (n = 2875)**		**P-value^b^**
	N	%	n	%	n	%	

**Demographic factors**							
Age							<0.0001
45–55	1,500	43.6	185	34.1	1,305	45.4	
55–65	909	26.4	153	28.2	751	26.1	
65+	1,030	30.0	205	37.8	819	28.5	
Education							<0.0001
None	997	29.0	223	41.1	767	26.7	
Elementary school or lower	1,446	42.1	201	37.0	1,239	43.1	
Junior high school	333	9.7	40	7.4	291	10.1	
Senior high school	418	12.2	57	10.5	358	12.5	
Undergraduate or graduate	243	7.1	21	3.9	219	7.6	
Missing	2	0.1	1	0.2	1	0.0	
Marital status							0.0138
Unmarried	951	27.7	174	32.0	773	26.9	
Married	2,488	72.4	369	68.00	2,102	73.1	
**Economic factors**							
Employment status							0.0001
Employed	1,046	30.4	117	21.6	919	31.9	
Unemployed	2,389	69.5	424	78.1	1,956	68.0	
Missing	4	0.1	2	0.4	0	0	
Personal income (NT $)							0.0005
None	1,448	42.1	270	49.7	1,173	40.8	
<4.999	494	14.4	83	15.3	409	14.2	
5,000~9,999	269	7.8	34	6.3	234	8.1	
10,000~14,999	269	7.8	42	7.7	223	7.8	
15,000~19,999	261	7.6	33	6.1	227	7.9	
20,000~39,999	418	12.2	46	8.5	367	12.8	
>= 40,000	243	7.1	27	5.0	215	7.5	
Missing	37	1.1	8	1.50	27	1.0	
**Health Status**							0.1672
Illness in the past six months							
No	757	22.0	107	19.7	643	22.4	
Yes	2,680	78.9	436	80.3	2,230	77.6	
Missing	2	0.1	0	0	2	0.1	

^a^21 missing without consent choice; P-value^b^: chi-square test

We also reviewed the participant's January 1, 2002 to December 31, 2002 NHI outpatient claims. Out of the 2875 female participants, 504 had NHI records indicating they had sought treatment for menopause related problems during the study period starting six months before and ending six months after the WHI report was published.

### Measures

In this study, the dependent variable was whether or not MHT was prescribed during outpatient visits for menopause-related symptoms. A menopause-related visit was defined if the patient was diagnosed as having a menopausal condition represented by one of the seven ICD9CM codes: 627.0 menopausal menorrhagia, 627.1 postmenopausal bleeding, 627.2 menopausal or female climacteric states, 627.3 postmenopausal atrophic vaginitis, 627.4 states associated with artificial menopause, 627.8 other specified menopausal & postmenopausal disorders, or 627.9 unspecified menopausal & postmenopausal disorder. We created a dummy variable to indicate whether or not MHT was prescribed: 1, if estrogen was prescribed; zero, if not.

Independent variables included the effect of WHI report, variables related to outpatient visits for menopause and variables of patient demographics. A dummy variable was created to indicate whether the WHI report had been released. A value of 1 was assigned if an outpatient visit occurred on or after July, 2002 and a value of 0 if a visit occurred during the first half of 2002. Variables related to outpatient visits for menopause, including the categories of health care institution and physician specialty, were mainly extracted from NHI outpatient records. Health care institutions were classified into four accreditation levels: academic medical centers, metropolitan hospitals, local community hospitals, and physician clinics. A dummy variable was assigned to physician specialty, 1 if the patient was treated by an obstetrician or a gynecologist, and 0 if treated by a physician of any other medical specialty. Patient demographic data, including age, marital status, employment status, and monthly income, were obtained from the 2001 NHIS. Patients were categorized into the following three age groups: 45–54, 55–64, 65 and above. Dummy variables were also created for marital status, employment status: 1 indicating yes, and zero indicating no. Personal monthly income was divided into five categories: no income, less than NT $5000, $5000 – 19999, $20000 – 39999 and NT $40,000 or above.

Two dummy health status variables were also created. The first dummy health status variable, which represented whether the respondent experienced illnesses during the six-month period leading up to the interview, was extracted from the 2001 NHIS. A value of 1 was assigned if any kind of illness had been experienced, and a value of 0 if not. Another dummy health status variable, representing whether the patient had been diagnosed as having osteoporosis, was extracted from the NHI outpatient record. A value of 1 was assigned if the patient had been diagnosed or treated for osteoporosis or osteoporosis-related condition (ICD9CM = 733.00 osteoporosis, unspecified, 733.01 senile osteoporosis, 733.02 Idiopathic osteoporosis, 733.03 disuse osteoporosis, 733.09 other osteoporosis), and a value of 0 if not.

### Statistical analysis

Our unit of analysis was 2,549 outpatient visits for menopause-related symptoms of 504 study participants. Our reference group included women visiting outpatient visits for menopause-related symptoms before the WHI report. Logistic regressions were used to analyze the effect of the WHI report and women's characteristics such as age, educational level, marital status, employment status, personal monthly income, illness during the previous six months and osteoporosis on the likelihood that MHT was prescribed in the outpatient visit for menopause-related symptoms. To reduce the effect of repeated measurements, we estimated logistic regressions using random-effect model. Data management and logistic regression model used in our study was done using SAS Version 8.0 (Chicago, Illinois).

In addition, we introduced interaction terms of WHI with these two variables in another two logistic regressions: one with WHI interacting with medical institution characteristics, and another one with WHI interacting with women's educational levels. The interaction models were used to explore the association of the period (before and after the release of WHI report) and the prescription of MHT in different levels of education and accreditation levels of medical facility. A linear function of the coefficient was estimated and tested in these models with the interaction terms. Other covariates were also included in these models. However, in order to simplify interpretation, we showed the effect of publication of the WHI on the four types of hospitals instead of the interaction terms. Also, we showed the effect of publication of the WHI on the five levels education instead of the interaction terms.

## Results

The NHI records of 504 participants indicated that they had sought treatment for menopause related problems during the study period starting six months before and ending six months after the WHI report was published (Table 2). Of these 504 women, 28.6% (n = 144) had NHI outpatient records for menopause only during the six-month period leading up the release of WHI report, 51.2% (n = 258) before and after, and 20.2% (n = 102) during the 6-month period following the WHI period only (Table 2). Of the 402 women who had outpatient visits for menopause related problems both before the WHI report, 361 (89.8%) of them had at least one outpatient record with MHT prescription, 288 (80%) of 360 women, who had NHI outpatient visits for menopause six months after the WHI report, had at least one outpatient record with a MHT prescription.

**Table 2:** 504 females aged 45 years or above with outpatient visits for menopause

	**Number of individuals having outpatient visits for menopause (N = 504)**					
	
	**Total (N = 504)**		**Before WHI (N = 402)**		**After WHI (N = 360)**	
	**N**	**%**	**N**	**%**	**N**	**%**

**Demographic factors**						
**Age**						
45–55	292	57.9	232	57.7	214	59.4
55–65	142	28.2	111	27.6	103	28.6
65+	70	13.9	59	14.7	43	11.9
**Education**						
None	70	13.9	53	13.2	48	13.3
Elementary school or lower	245	48.6	194	48.3	170	47.2
Junior high school	63	12.5	56	13.9	48	13.3
Senior high school	83	16.5	63	15.7	61	16.9
Undergraduate or graduate	43	8.5	36	9.0	33	9.2
Missing	0					
**Marital status**						
Unmarried	92	18.3	76	18.9	60	16.7
Married	412	81.8	326	81.1	300	83.3
**Economic factors**						
**Employment status**						
Employed	169	33.5	133	33.1	120	33.3
Unemployed	335	66.5	269	66.9	240	66.7
**Personal income (NT $)**						
None	197	39.1	157	39.1	139	38.6
<4,999	56	11.1	44	11.0	38	10.6
5,000~19,999	104	20.6	86	21.4	79	21.9
20,000~39,999	87	17.3	64	15.9	64	17.8
>= 40,000	55	10.9	47	11.7	36	10.0
Missing	5	1.0	4	1.0	4	1.1
**Health Status**						
Illness in the past six months						
No	76	15.1	65	16.2	48	13.3
Yes	428	84.9	337	83.8	312	86.7
Osteoporosis						
No	353	70.0	294	73.1	274	76.1
Yes	151	30.0	108	26.9	314	23.9

**#Total outpatient visits for**	**2549**		**1392**		**1157**	
**menopause-related symptoms**						
Proportion of visits prescribing	1999	78.4	1155	83.0	844	73.0
MHT						
Proportion of visits prescribing						
MHT (stratified by type of institution)						
Academic medical center	475	58.7	278	65.2	196	49.0
Metropolitan hospital	627	82.9	342	84.8	285	80.7
Local community hospital	492	81.1	244	87.7	248	74.6
Physician clinic	955	83.9	527	89.0	428	77.6
Proportion of visits served by obstetricians or gynecologists	1295	50.8	712	51.2	583	50.4

#: Of these 504 women, 28.6% (n = 144) had NHI outpatient records for menopause only during the six-month period leading up the release of WHI report, 51.2% (n = 258) before and after, and 20.2% (n = 102) during the 6-month period following the WHI period only

Most of the 504 study participants, who had outpatient visits for menopause during our study period, were aged 45 – 55 years old (57.9%), had elementary school educations or lower (48.6%), were married (81.8%), were not employed (66.5%) or had no personal income (39.1%). Of these 504 females, most (84.9%) had experienced an illness during the six-month period leading up to the interview, and most (70.0%) had no NHI outpatient records for osteoporosis. In total, these 504 women made 2549 outpatient visits for menopause-related symptoms, 50.8% were being treated by either an obstetrician or gynecologist and 79.3% had been prescribed MHT. The proportion of outpatient visits in which MHT was prescribed dropped from 83.0% (n = 1392) before WHI to 73.0% (n = 1157) after WHI.

Table 3 shows the results of logistic regressions of random effect. Model 1 included the dummy variable of WHI effect without interaction terms; Model 2 included the interaction of WHI and women education; and Model 3 included the interaction of WHI and the accreditation level of medical institution. Overall, based on Model 1, the likelihood that MHT would be prescribed during an outpatient visit decreased after the WHI report (OR 0.36, 95%CI 0.25 to 0.52). Hospitals not classified as academic medical centers were more likely to prescribe MHT than academic medical centers (OR from 14.87 for metropolitan hospital to 26.42 for physician clinic). Obstetricians or gynecologists were more likely to prescribe MHT than physicians of other specialties (OR 5.34, 95% CI 3.45–8.26). Marital status, employment status, personal income, the diagnosis of osteoporosis, or illness was not found to be associated with the prescription of MHT the six months leading up to the interview. The impact of the WHI report on the prescription of HRT analyzed by level of education and the accreditation level of medical care institution was estimated in the two interaction models, Model 2 and Model 3. Women with college level educations or higher were less likely to be prescribed MHT after the WHI report than before (Model 2; OR 0.30; 95% CI 0.11–0.83). Academic medical centers were less likely to prescribe MHT than other medical care institutions after the release of WHI report (Model 3; OR 0.15; 95% CI 0.34–0.63).

**Table 3:** Logistic regressions of random effect for MHT treatment in menopause outpatient visits

	**Outpatient visits (% prescribing MHT)**		**Model 1 – no interaction**	**Model 2 – interaction of WHI and woman education**	**Model 3 – interaction of WHI and hospital level**
	n	%^a^	Odds Ratio (95% CI)	Odds Ratio (95% CI)	Odds Ratio (95% CI)

**WHI report announcement**					
After	1157	72.95	0.36 (0.25–0.52)		
Before (reference group)	1392	82.97			
					
**Medical care institution**					
Metropolitan hospital	627	82.93	14.87 (7.28–30.37)	11.98 (5.82–24.68)	7.02 (3.23–15.27)
Local community hospital	492	81.10	17.00 (7.92–36.49)	12.09 (5.81–25.14)	12.13 (4.84–30.41)
Physician clinic	955	83.87	26.42 (13.34–52.32)	16.52 (8.43–32.39)	18.22 (8.12–40.89)
Ref: Academic medical center	475	58.74			
					
**WHI on Medical care institution^b^**					
WHI effect on metropolitan hospital					6.25 (2.08–18.72)
WHI effect on local community hospital					1.69 (0.52–5.49)
WHI effect on physician clinic					2.13 (0.76–5.98)
WHI effect on academic medical center					0.15 (0.34–0.63)
					
**Physician specialty**					
Obstetrician or gynecologist	1295	85.10	5.34 (3.45–8.26)	6.26 (3.92–10.00)	5.56 (3.60–8.58)
Ref: other specialty	1254	71.53			
					
**Demographic factors**					
**Age**			0.73 (0.41–1.32)	0.72 (0.37–1.40)	0.66 (0.36–1.20)
55–65	719	75.52			
65+	348	83.62	2.33 (0.81–6.69)	2.43 (0.86–6.92)	4.38 (1.41–13.54)
Ref: 45–55^+^	1482	78.61			
**Education**					
Senior high school	373	75.87	0.53 (0.21–1.35)	0.33 (0.09–1.21)	2.46 (1.02–5.93)
Junior high school	446	78.03	0.54 (0.19–1.55)	0.38 (0.11–1.31)	3.11 (1.19–8.10)
Elementary school or lower	1180	80.76	0.95 (0.41–2.19)	0.62 (0.22–1.80)	4.50 (1.83–11.04)
No	322	79.19	1.18 (0.43–3.20)	1.10 (0.31–3.84)	4.90 (1.75–13.72)
Ref: Undergraduate or graduate	228	70.18			
					
**WHI effect on woman education^c^**					
WHI effect on women with senior high school				1.64 (0.43–6.22)	
WHI effect on women with junior high school				1.10 (0.29–4.16)	
WHI effect on women with elementary school or lower				1.28 (0.40–4.08)	
WHI effect on women with no education				0.85 (0.20–3.53)	
WHI effect on women with undergraduate or graduate				0.30 (0.11–0.83)	
					
**Marital status**					
Married	2141	77.11	1.03 (0.51–2.10)	0.64 (0.34–1.22)	1.18 (0.62–2.23)
Ref: Unmarried	408	85.29			
					
**Economic factors**					
**Employment status**					
Employed	841	79.31	0.94 (0.45–1.94)	0.37 (0.17–0.76)*	0.63 (0.30–1.30)
Ref: Unemployed	1708	77.99			
**Personal income (NT $)**					
<4,999	293	78.16	0.73 (0.31–1.72)	0.66 (0.28–1.59)	0.76 (0.31–1.89)
5,000~19,999	562	79.89	0.72 (0.30–1.71)	0.36 (0.28–1.59)	0.74 (0.32–1.73)
20,000~39,999	431	79.35	1.20 (0.57–2.55)	0.39 (0.18–0.85)	1.14 (0.53–2.45)
>= 40,000	259	71.43	0.24 (0.09–0.59)	0.33 (0.13–0.85)	0.86 (0.31–2.39)
Ref: None	993	79.15			
					
**Health Status**					
Illness in the past six months					
Yes	2188	78.11	1.08 (0.49–2.39)	2.18 (1.07–4.47)	0.81 (0.38–1.73)
Ref: No	361	80.33			
Osteoporosis					
Yes	741	80.43	1.63 (0.91–2.89)	1.48 (0.84–2.59)	1.37 (0.80–2.33)
Ref: No	1808	77.60			

%^a^: Percentages in the second column of table 3 were the proportion of outpatient visits with a prescription of MHT in each category. For example, among the 1,157 outpatient visits after the publication of WHI report, there're 72.95% (844 visits) with a prescription of MHT.

WHI and Medical care institution^b^: A linear function of the coefficient was estimated and tested in Model 3 with the interaction terms. In order to simplify interpretation, we showed the effect of publication of the WHI on the four types of hospital instead of the interaction terms.

WHI effect and woman education^c^: A linear function of the coefficient was estimated and tested in Model 2 with the interaction terms. In order to simplify interpretation, we showed the effect of publication of the WHI on the five categories of level of women education instead of the interaction terms.

## Discussion

MHT was once widely thought to be an effective treatment for menopause-related symptoms. In year 2002, the WHI trial of estrogen plus progestin in postmenopausal women found that overall health risks of this therapy outweighed the benefits for these women [7]. The release of that report provides us the opportunity to study to what extent publicizing new scientific evidence changes public acceptance of certain modes of therapy and health care provider's choice of therapeutic regimens, in this case menopausal hormone therapy.

In this study, as other studies have indicated, the WHI report led to a significant decline on the use of MHT for menopausal difficulties [12-16]. Like other studies [29-32], we found that gynecologists were more likely to prescribe MHT than physicians with other medical specialties, but we also found that the decision whether MHT would be prescribed was not totally decided by physicians alone, but had also to do with who was seeking therapy. Women with college level educations or higher were less likely to be prescribed MHT than the women with high-school level educations or lower and health care providers were found to vary considerably in whether MHT was prescribed.

One of the limitations of this study is its focus on the MHT for the treatment of menopausal-related symptoms. We did not take into account that some women might stop seeking western medicine treatment at outpatient visits for menopausal-related symptoms and start searching for other non-pharmacological treatments (e.g. herbal products or health supplements). Since our data source was obtained from the NHI outpatient visit records, no information on over-the counter herbal products or nutritional supplements for menopausal symptoms was included. Thus, our findings may underestimate women's reaction to the WHI report. Nevertheless, our sample was drawn from a nationwide interview survey and linked to NHI claim data, allowing us to obtain real utilization information and identify the co-existing condition of osteoporosis. Data collected this way is superior to self-reported information, which can be subject to recall bias. 98.3% of the people we surveyed were insured under the NHI program, with almost all of their medical costs covered through this program. This lends credence to our findings, in that financial barriers to MHT were non-existent.

Another limitation of our study is potential selection bias. The sample base for this study was drawn from the women who were interviewed in the 2001 National Health Interview Survey and aged 45 years or above. Only those who signed the inform consent for linking NHI claim data would be included in this study. Those who did not sign the inform consent were more likely to be older, less educated, unmarried, unemployed, have lower personal income and be healthier. Our logistic regression predicted that women who were unemployed and less educated women were more likely to be prescribed MHT. Thus, we would suspect that those women who were not included in this paper were those who were more likely to be prescribed MHT. After the WHI report, women with undergraduate or graduate (higher education) were less likely to receive MHT OR = 0.30 (0.11–0.83). Again, we suspect that the effect of WHI on reducing prescription of MHT may be somewhat over-estimated, because lower-educated women were more likely to be excluded and less responsive to WHI report than higher-educated women.

Finally, this study was undertaken only six months following publication of the WHI report. In order to keep track of stable changes in the patterns of practice patterns that take place after publication of such an article, longer follow-up periods may be necessary, especially of subgroups of women such as women with previous hysterectomies, those older than 65, and women living in areas of high social deprivation. Future research should consider factors leading to the effective dissemination of new clinical trial findings for women.

## Conclusion

The WHI report brought about a substantial decline in the prescription of MHT to treat menopause-related symptoms in Taiwan. The decision whether to prescribe MHT for menopause-related symptoms was not purely dominated by the physician. Women with college level educations or higher were less likely to be prescribed MHT than the women with high-school level education or lower after the release of WHI report, and academic medical centers were less likely and metropolitan hospitals more likely to prescribe MHT than medical providers with different accreditations after the release of WHI report.

## Competing interests

Dr. WF Huang received a research grant from Merck Foundation between October 1, 2003 and September 30, 2004 for a project exploring the development of biotechnology and incentive strategies in Taiwan. He also represented the government on the board of directors of two biotech companies in Taiwan, for which he received a nominal fee until July 31^st^, 2005. However, this paper involves no competing interest at all. None of the other co-authors of this manuscript, Ms. FY Hsiao and WC Liu, and Dr. YW Tsai, had possible competing interest to declare.

## Authors' contributions

WFH, the original initiator of this study concept, was responsible for organizing the research group, coordinating the study efforts, assuring quality of methods, data retrieval, processing, and interpretation of the results. He furnished the draft of this manuscript and submitted it for consideration for publication.

FYH participated in the study design, helped carry out data retrieval and processing, and the statistical analysis. She drafted the initial English copy of this manuscript.

YWT participated in the design of this and methodology it used. She contributed to the analytical framework, the interpretation of study results, and gave critical comments on the draft. She was also responsible for quality assurance of software programming and data quality.

WCL retrieved the NHI database and linked to NHIS. She performed the data processing, statistical analysis, and participated in preparing the draft of this manuscript. All authors read and approved the final manuscript.

## Pre-publication history

The pre-publication history for this paper can be accessed here:


